# Urolithin A protects against acetaminophen-induced liver injury in mice via sustained activation of Nrf2

**DOI:** 10.7150/ijbs.69116

**Published:** 2022-02-28

**Authors:** Zhimin Gao, Wei Yi, Junyuan Tang, Yuling Sun, Jianrong Huang, Tian Lan, Xiaoyan Dai, Suowen Xu, Zheng-Gen Jin, Xiaoqian Wu

**Affiliations:** 1 Key Laboratory of Molecular Target & Clinical Pharmacology and the State & NMPA Key Laboratory of Respiratory Disease, School of Pharmaceutical Sciences& The Fifth Affiliated Hospital, Guangzhou Medical University, Guangzhou511436, China; 2 Institute of Chinese Medicine, Guangdong Pharmaceutical University, Guangzhou 510006, China; 3 Department of Endocrinology and Metabolism, The First Affiliated Hospital, Division of Life Sciences and Medicine, University of Science and Technology of China (USTC), Hefei 230037, China; 4 Aab Cardiovascular Research Institute, Department of Medicine, University of Rochester School of Medicine and Dentistry, Rochester, NY, USA

**Keywords:** Acute liver injury, Acetaminophen, Mitophagy, Nrf2, Urolithin A

## Abstract

Acetaminophen overdose is a leading cause of acute live failure worldwide. N-acetylcysteine (NAC), as the only antidote, is limited due to its narrow therapeutic time window. Here we demonstrated that Urolithin A (UA), a metabolite of ellagitannin natural products in the gastrointestinal flora, protected against acetaminophen-induced liver injury (AILI) and is superior to NAC in terms of dosage and therapeutical time window. Transcriptomics assay revealed that UA promotes mitophagy and activated Nrf2/ARE signaling in the liver. Consistent with that, mitophagy and Nrf2/ARE signaling were activated, with less oxidative stress in UA-treated liver. Subsequently, molecular docking and dynamics simulation study revealed a binding mode between UA and Nrf-2/Keap1 including the hydrogen-bonding network among oxygen atoms in UA with the Nrf-2/Keap1 residues Arg 415, Ser 508 and Ser 602, which in turn trigger Nrf2 nuclear translocation, subsequently leading to activation of Nrf-2 target genes (HO-1, NQO1). Of note, mitophagy inhibition failed to prevent the protection of UA against AILI, which instead was compromised with Nrf2 gene silencing both *in vivo* and *in vitro*. Collectively, our data indicate that UA alleviated acetaminophen-induced oxidative stress and hepatic necrosis via activating Nrf2/ARE signaling pathway, highlighting a therapeutical potential of UA for AILI.

## Introduction

Acetaminophen (APAP) is currently one of the most common antipyretic-analgesic and anti-inflammatory drugs worldwide 1. APAP is commonly considered to be safe at a dosage ranging from 325 to 650 mg every 4-6 hours, with a daily maximum of 4 g for an adult 2. However, APAP overdose-induced hepatotoxicity is a leading cause of acute liver failure worldwide 3, 4. Decades of research work have promoted an enriched understanding of APAP-induced liver injury (AILI). The anti-toxic phase II enzymes are saturated in the condition of APAP overdose, and the extra APAP will be metabolized into the highly reactive intermediate *N*-acetyl-*p*-benzoquinone imine (NAPQI) 5. As a result, excessive accumulation of NAPQI depletes glutathione (GSH) and binds with intracellular proteins covalently to form APAP protein adducts 6, which in turn result in mitochondrial dysfunction, reactive oxidative stress (ROS), and ultimately hepatocyte necrosis and apoptosis 7. Up to date, N-acetylcysteine (NAC) is the only standard detoxification drug approved for the treatment of AILI. NAC acts mainly via replenishing GSH to detoxicate NAPQI, which in turn reducing ROS at a relatively early stage. However, it is not always effective in patients with APAP toxicity at a late stage due to the therapeutic time limit 8, 9. Thus, novel strategies that can extend the therapeutic time frame for better survival are of urgent need.

Although the exact machinery by which APAP and its metabolites result in cellular damage is still unclear, it has been conferred that APAP induced AILI most probably stem from cumulative and additive effects of oxidative stress, mitochondrial dysfunction 10, inflammation 11, endoplasmic reticulum stress [12]and autophagy 13. The oxidative stress, which is derived from mitochondrial damage, leads to mitochondrial permeability transition pore opening and mitochondrial membrane potential (ΔΨm) loss, and even necrotic cell death characterized by HMGB1 release from the nuclei 14. In this regard, elucidating the mechanism of oxidative stress is imperative for discovering novel therapy strategies for APAP overdose.

Urolithins, a class of polyphenols, are naturally occurring metabolites originated by the gut microbiota after intake of foods rich in ellagitannins (ETs) and ellagic acid (EA) 15. In the species explored to date (including human beings), Urolithin D, C, A, and B are the sequential metabolites of EA, with increased lipophilicity 16, 17. Among the metabolites of ETs, Urolithin A (UA) is the most bioactive 18, 19. It was recently reported that UA enhances mitophagy and improves exercise capacity in two different models of age-related muscular dystrophy 19, 20. Moreover, a first in-human clinical trial revealed that UA was demonstrated biological safe and improved mitochondrial function in the elderly human 21. In this study, we aimed to investigate the therapeutic roles and potential molecular mechanism of UA in the treatment of APAP-induced hepatotoxicity.

## Materials and methods

### Animal experimental design

All animal experiments were performed in accordance with the Guide for the Care and Use of Laboratory Animals published by the United States National Institutes of Health (NIH publication no.8023, revised 1978). The protocol was approved by the Institutional Animal Care and Use Committee, Guangzhou Medical University, Guangzhou, China. Male C57BL/6J (6 to 8 weeks old) were purchased from Medical Experimental Animal Center of Guangdong Province. Atg5 *^flox/flox^* mice were purchased from GemPharmatech Company (Jiangsu, China) as described previously 22. Hepatocyte-specific Atg5 haploinsufficiency were generated by breeding Atg5 *^flox/flox^* mice with mice expressing the hepatocyte specific Albumin promoter-driven *cre* recombinase gene mice. Mice were housed in conditions of temperature (23 ± 2 ^o^C), humidity (60% ± 5%) and 12 h light-dark cycle at the Center of Laboratory Animal, Guangzhou Medical University. They were received humane care with food and water available *ad libitum*.

The acetaminophen hepatotoxicity model was established as previously described 23. For the treatment experiments, the 70 mice were randomized into 7 groups: (i) vehicle control (0.9% saline with 1% DMSO), (ii) vehicle control + UA (50 mg/kg), (iii) APAP (500 mg/kg), (iv) APAP (500 mg/kg) + UA (50 mg/kg), (v) APAP (500 mg/kg) + UA (100 mg/kg), (vi) APAP (500 mg/kg) + UA (150 mg/kg), (vii) APAP (500 mg/kg) + NAC (300 mg/kg). Urolithin A (Sigma, USA) was dissolved in dimethyl sulfoxide (DMSO) and store at -20 ^o^C. The stock solution was diluted in 0.9% saline for intraperitoneal injection. The concentration of UA used in the *in vivo* experiment was 50 mg/kg if not mentioned. NAC and APAP (Sigma, USA) were dissolved in warm sterile 0.9% saline respectively before injection.

For the survival test, another 15 male mice per group were fasted overnight, and then were challenged with a loading dose of 750 mg/kg APAP by intraperitoneal (i.p.) injection, followed UA (50 mg/kg). The survival rates of mice were recorded every 8 hours until 48 h after APAP challenge.

For the post-treatment experiment, another 6 male mice per group were treated with UA (50 mg/kg) or NAC (300 mg/kg) intraperitoneally 2 h or 4h after APAP (500 mg/kg) injection. The tissue was harvested at 12 h after APAP challenge.

### Biochemical assay

Mice blood samples were collected and stood at room temperature for 30 min. Subsequently, the blood sample were centrifuged at 4 ^o^C, 1500 rpm/min for 15 min. The serum levels of ALT and AST were determined with a kit according to the manufacture's introductions (Jiancheng Bioengineering Institute, Nanjing, China).

### Histological analysis

The liver tissues were freshly collected and immersed in 4% paraformaldehyde (PFA) and fixed for 12 h at 4 ^o^C, dehydrated in series of graded ethanol, embedded in paraffin. Then, the liver tissues were cut into a thickness of 5 μm sections, which were stained with hematoxylin-eosin (H&E) staining to assess the liver pathological changes using Nikon microscopy (Nikon Instruments Inc, Japan). The necrotic areas in liver tissue were quantified by Image J.

### Hepatic reactive oxygen species (ROS) staining and Immunofluorescence staining

The freshly isolated liver tissues were immediately embedded in tissue-freezing medium (O. C. T compound, Sakura Finetek, Tokyo, Japan) and stored at -80 ^o^C. Frozen sections were cut at 8 μm thickness on a Leica CM1900 cryotome. For DHE staining, the section was incubated in 10 μM DHE (KeyGEN BioTECH, Nanjing, China) at 37 ^o^C for 10 min and then 10 μg/mL 4', 6'-diamidino-2-phenylindole (DAPI) was used to stain the nucleus. Images were acquired by fluorescent microscope with 200× magnification and analyzed by image J software.

For immunofluorescence staining, tissues and cells were fixed in 4% PFA. The frozen tissues were embedded in O.C.T compound and sectioned at 20 μm. After blocking and permeabilizing the samples with 0.3% Triton X-100 and 10% goat serum, cryosections were probed with the primary antibodies HMGB1 (Boster Biological Technology, Wuhan, China) and incubated overnight at 4^ o^C. The sections were then washed with PBS and stained with fluorescently labeled Alexa 488 conjugated secondary antibody for 1 h at room temperature. The stained sections were mounted with DAPI-containing mounting medium (Thermo Fisher Scientific, USA). Finally, images were viewed on Nikon fluorescence microscope and were analyzed using Image J software.

### Transmission electron microscopy (TEM)

As described previously 24, liver tissue was cut into sections of 1 mm^3^, and were fixed with 2.5% glutaraldehyde in phosphate buffer, washed and fixed in 1% OsO4, then dehydrated through graded ethanol solutions and embedded in Spurr resin. The sections (70 nm) were counterstained with uranyl acetate and lead citrate. Image were acquired with a transmission electron microscope (HITACHI H-600, Japan).

### mRNA sequencing

The mice were injected with APAP (500 mg/kg) or DMSO for 2 h, then followed by UA (50 mg/kg) or the Vehicle for another 6 h. After the treatment, the liver tissue was sequenced by the high-throughput sequencing service (IGE Biotechnology, Guangzhou, China). Total RNAs were obtained using a RNeasy Mini kit (Qiagen, Valencia, CA, USA) and purified using poly-T oligo-attached magnetic beads. The RNA concentration was determined with Nanodrop 2000 (Thermo Fisher Scientific). A total of 1 µg RNA was sequenced by Nova 6000 (Illumina, San Diego, CA, USA). *P* < 0.05 and fold change > 2) generated by DESeq2 were subsequently analyzed for enrichment of biological terms with the Database for Annotation, Visualization and Integrated Discovery (DAVID) bioinformatics platform. Hierarchical cluster analysis and volcano plots were performed to analyze the gene expression patterns. Gene Ontology (GO) enrichment analysis of differentially expressed genes was implemented by the cluster Profiler R package, in which gene length bias was corrected. KEGG pathway enrichment analysis of the differentially expressed transcripts was performed using R based on the hypergeometric distribution.

### Cell culture and cell viability/cytotoxicity assay

Human non-tumor hepatic L02 cells, obtained from Type Culture Collection of Chinese Academy of Sciences (Shanghai, China), were cultured in Roswell Park Memorial Institute (RPMI) 1640 supplemented with 10% fetal bovine serum, 2 mM glutamine at 37 ℃ with 95% air and 5% CO_2_. Cell viabilities were determined using CCK8 assay kit (Hanbio Biotechnology, Shanghai, China) according to the manufacture's instruction. L02 cells were seeded at a density of 1 × 10^4^ cells / well in 96-well plates for 24 h. The cells were treated with UA at final concentrations of 1 μM, 5 μM, 10 μM in the presence or absence of APAP (10 mM) for 24 hours. A 10 μL of CCK8 solution was added to each well, and the plates were incubated at 37 °C for 1 h in the dark. The optical density of each well was measured at 450 nm using the microplate reader. The level of the lactate dehydrogenase (LDH) in the cell culture supernatant was determined according to the manufacturer's instructions using a commercial kit (Jiancheng Bioengineering Institute, Nanjing, China). UA at concentration of 5 μM was used if not mentioned thereafter for the *in vitro* experiment.

### GFP-LC3 adenovirus transfection and live cell staining

The GFP-LC3 adenovirus were prepared by Hanbio Technology Corporation (Shanghai, China). The L02 cells were seeded 24 h prior to transduction. As described previously 24, cells were transfected with adenoviral particles at a MOI of 50 for 36 h before treatment. At the end of experiment, Mito-Tracker Red (50 nM, Thermo Fisher Scientific, Rockford, IL, USA) or lysotracker (100 nM, Thermo Fisher Scientific, Rockford, IL, USA) were added to the culture medium and incubated at 37 °C for 30 mins in the dark. After rinsing with PBS for three times, the fluorescent puncta in cells were recorded using Nikon A1R confocal microscopy. The average number of LC3 puncta was determined by counting of fluorescent puncta from at least 3 individual experiments. At least 40 cells were scored in each experiment. The numbers of mitochondria co-localized with lysosome were quantified as described previously 25.

### Adeno-associated virus serotype 8 (AAV8)-based liver-specific knockdown of Nrf2

To generate a liver-specific knockdown of *Nrf2*, *Nrf2*-specific small short hairpin RNA (shRNA) was cloned and packaged into an adeno-associated virus 8 (AAV8) with the liver-specific thyroxin binding globulin (TBG) promoter, which were designed by GeneChem (Shanghai, China). In brief, male C57BL/6 mice were injected through the tail vein with 30 μL of AAV8-shRNA-Nrf2 or the Scramble virus suspension (virus titer 5 × 10^11^ vg/mL) blended with 30 μL phosphate-buffered saline. One week after AAV infection, the mice were challenged with APAP injection with or without UA treatment (50mg/kg). The liver tissue and serum were harvested at 12 h after APAP challenge.

### siRNA transfection

L-02 cells were cultured for 24 h and then 50 nM negative control small interference RNA (NC siRNA) or Nrf2 siRNA (RiboBio, Guangzhou, China) were transiently transfected using lipofectamine RNAiMAX (Thermo Fisher Scientific, Waltham, USA). After siRNA transfection for 48 h, the cells were treated with or without UA (5 μM) in the presence or absence of APAP (10 mM) for 24 h.

### Protein extract and Western Blot Analysis

As described previously 25, the nuclear and cytoplasmic extracts were prepared using a Nuclear and Cytoplasmic Extraction Reagents kit (BestBio, Shanghai, China) in accordance with the manufacturer's instructions. Then the protein extracts concentration was determined using the BCA protein concentration assay kit (Thermo Fisher Scientific, Waltham, USA). Protein (equal concentration) samples were separated by running on a 10% SDS-PAGE gel and transferred onto a PVDF membrane. The PVDF membrane was blocked and then followed by incubation with the primary antibody at 4 °C overnight. The next day, the membrane was incubated with the anti-rabbit secondary antibody for 1 h at room temperature. The protein bands were detected by chemiluminescence using the 20 × LumiGLO Reagent Cell Signaling Technology and the intensity was calculated by densitometry.

### Molecular docking

Molecular docking was carried out with Discovery Studio 3.1 to get the starting structure of Nrf2/UA for further simulation. The Nrf2/Keap1 complex crystal structures were obtained from the Protein Data Bank database (PDB ID: 1X2R). Brifely, the Protein Preparation Wizard in Discovery Studio 3.1 suite was used for the treatment of the protein structure with all bond orders reassigned, hydrogen atoms added and water molecules deleted. Next, the minimization of the sampled hydrogens was conducted to realize the optimization of the hydrogen-bonding network. Finally, a restrained minimization was further performed by virtue of the CHARMm force field. In addition, by sequentially adding explicit hydrogen atoms, applying the CHARMm force field and performing geometry optimization with the prepared ligands module of Discovery Studio 3.1, the UA was rationally prepared. The docking grid was generated accordingly, in which its center was set to the native ligand and the grid size was set similarly (The search grid of binding site was identified as center_x: 9.0235, center_y: 66.3648, and center_z: -10.7141 with the radius of 11.15 Å). Afterwards, UA was subjected to the docking at the extra precision (XP) level (CDOCKER).

### Molecular dynamics simulation

The GROMACS Package (version 2019.03) was used for MD simulations, and 1X2R was prepared with the force field amber99sb.ff 26. On the basis of the results from CDOCKER module in Discovery Studio 3.1, we chose the best docking pose as the initial confirmation for further simulation. The AnteChamber Python Parser Interface (ACPYPE) was used to parameterize the substrate. Both the substrate and protein complex were placed in a periodic box and the minimum distance between the system and boundary of the box was 15 Å. The system, which contained 6044 H_2_O, 25 Na^+^ and 16 Cl^-^, was solvated with atomistic TIP3P water in an octahedral water box and neutralized by adding 0.150 M chloride and sodium ions. The system energy was minimized using the steepest descent minimization method by 50,000 steps, and the positions of heavy atoms were restricted to run both NVT and NPT equilibration by 50,000 steps. The temperature was kept at 300 K and the pressure was set at 1 bar. The system is well equilibrated at the desired temperature and pressure upon the two equilibration phases completing. Generally, a 100 ns unrestrained simulation was carried out, during which the energy and coordinate system of the trajectory were saved per 10 ps. The trajectory was generated by the GROMACS trjconv command after the MD simulations. The root-mean-square deviation (RMSD) values of 1X2R and substrates complexes were obtained via the GROMACS rms command. The interactions between the substrates and 1X2R were analyzed by Discovery Studio 3.1 and visualized with PyMOL (version 2.5).

### Statistical analysis

All data were expressed as means ± SEM and analyzed with the statistical software GraphPad Prism 8.0 (GraphPad Software Inc, San Diego, CA, USA). For multiple comparisons, one-way analysis of variance followed by with Bonferroni post hoc test was used. *P* < 0.05 was considered statistically significant.

## Results

### Urolithin A mitigates APAP-induced hepatic injury in the mice

**Figure 1:**
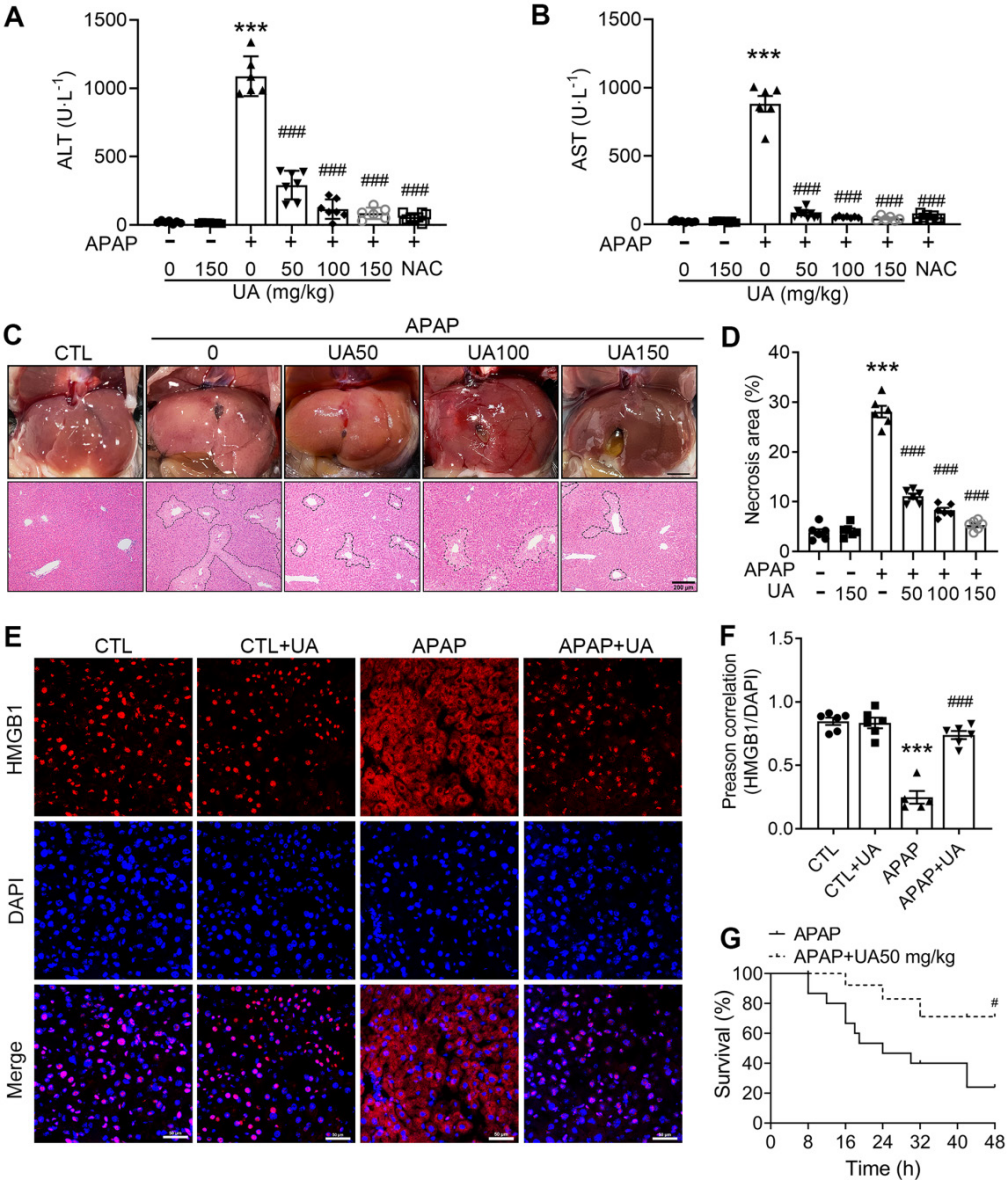
** UA Protected against Acetaminophen-induced liver injury *in vivo***. **(A)** Serum ALT and **(B)** AST activities in mice with various concentrations of Urolithin A in the presence or absence of APAP administration (500 mg/kg), *n* = 6. **(C)** The representative image of liver tissue (scale bar: 500 mm) and representative image of H&E-stained liver sections at 100 × magnification, scale bar: 200 μm. APAP-induced centrilobular necrosis was indicated by the dotted line. **(D)** The quantification of necrosis area of the liver tissue, *n* = 6. **(E)** HMGB1 staining of liver sections (scale bar: 50 μm). **(F)** Quantification of colocalization of HMGB1 and nuclei by Image J (NIH, Bethesda, MD) software,* n* = 6. **(G)** The survival rate in the APAP challenged (750 mg/kg) mice with or without UA treatment (15 mice per group). Data were represented as the means ± SEM. ^***^ *P* < 0.001 *vs.* CTL; ^#^ *P* < 0.05, ^###^ *P* < 0.001 *vs.* APAP.

To explore the therapeutical effect of UA on AILI, the C57BL/6J mice were administrated with 500 mg/kg APAP by intraperitoneal injection, followed by different concentrations of UA (50,100,150 mg/kg) treatment (Figure S1A). The serum ALT and AST, both of which are well-established markers of liver injury, were obviously raised in the mice with APAP injection (Figure 1A and B). Gross morphological view of liver tissue showed that APAP overdose caused dotted pattern and severe signs of hemorrhagic bleeding. Histological assessment of H&E staining displayed distinctive bridging necrosis within the centrilobular region of the liver in the APAP challenged mice (Figure 1C and D). Notably, APAP-induced increase of serum ALT and AST as well as the hepatic necrosis were significantly attenuated by different concentrations of UA (Figure 1A-D). Moreover, immunostaining of the liver specimen revealed that HMGB1 translocated to the cytoplasm in the APAP overdosed mice, an indicative of nuclear cell death^27^, which was significantly blunted by UA treatment (Figure 1E and F).

In addition, we examined the effect of UA on APAP-induced mortality by a lethal dose of APAP (750 mg/kg) intraperitoneally. The survival rate of the mice was monitored every 8 hours until 48 h after drug administration. During this observation period, UA treatment significantly increased the survival of the mice. The mortality of the vehicle treated mice is over 50%, whereas over 70% of mice with Urolithin A treatment survived at 24 h after APAP challenge (Figure 1G). Taken together, these data suggest that UA renders mice more resistant to APAP-induced AILI.

### Urolithin A protected against Acetaminophen-induced cytotoxicity *in vitro*

**Figure 2:**
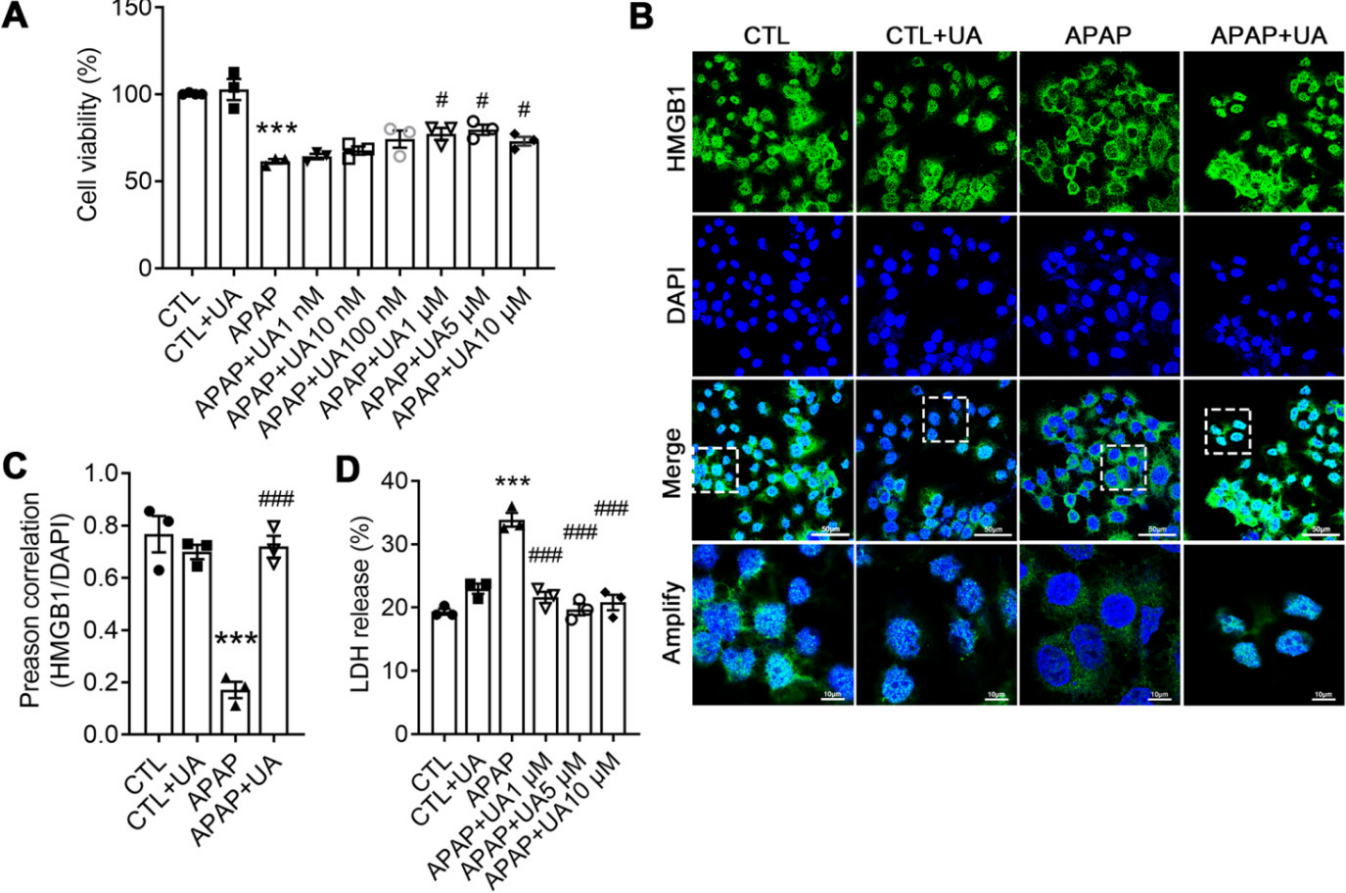
** UA Protected against Acetaminophen-induced Cytotoxicity *in vitro***. **(A)** L02 hepatocytes were subjected to UA at different concentrations with or without APAP (10 mM) for 24 h. Cell viability was determined by CCK8 assay.** (B)** HMGB1 (green) staining of L02 cells. Representative images with amplification indicating example cells with cytoplasmic HMGB1 staining and hollow nuclei (scale bars: 50 μm for upper and 10 μm for bottom), and quantification of colocalization of HMGB1 and nuclei by Image J software in at least 20 randomly selected individual cells. **(C)** Quantification of LDH released into the culture medium of L02 cells. Data were represented as the means ± SEM, the experiment was repeated at least 3 times, ^***^ *P* < 0.001 *vs.* CTL; ^#^ *P* < 0.05, ^###^ *P* < 0.001 *vs.* APAP.

We next examined the role of UA in APAP-triggered hepatocyte toxicity in an *in vitro* setting to exclude *in vivo* unknown factors. As shown in Figure 2A, UA significantly suppressed APAP-induced cytotoxicity in a concentration-dependent manner. Immunostaining revealed that UA mitigated HMGB1 cytoplasmic translocation induced by APAP (Figure 2B). In addition, the release of lactate dehydrogenase (LDH), a necrotic marker, due to APAP injury was also alleviated significantly after UA treatment (Figure 2C). Therefore, these findings confirmed that UA protected against APAP-induced cytotoxicity* in vitro*.

### Urolithin A promoted Mitophagy with APAP overdose both *in vivo* and *in vitro*

**Figure 3:**
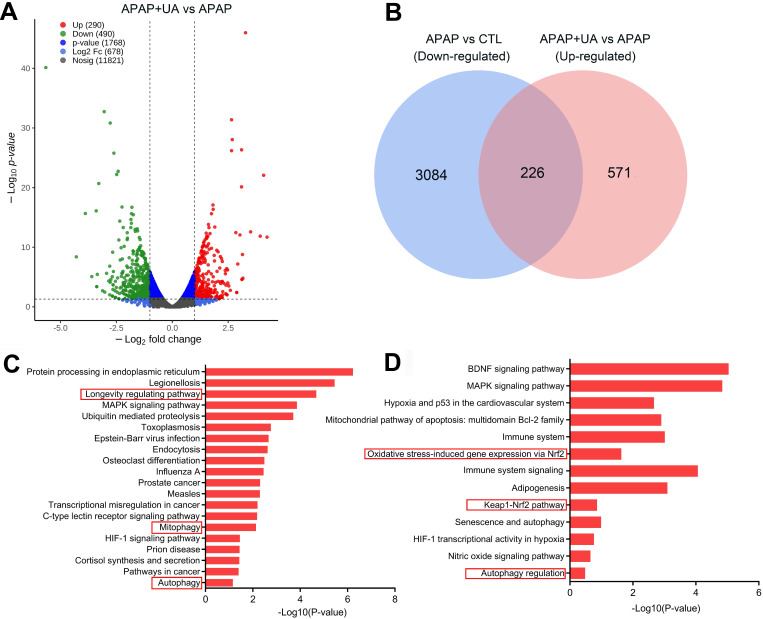
**RNA-Seq identifies transcriptome that are regulated by UA in the APAP-challenged liver tissue. (A)** Volcano plot shows magnitude and significance of genes that altered in the UA treated liver versus vehicle treated liver after APAP challenge. Genes of downregulation (left) and upregulation (right) in APAP+UA versus APAP were plotted in green and red dot, respectively. **(B)** Venn diagram analysis showed that 226 genes were overlapped based on the differentially expressed genes that are downregulated in the APAP vs. Control (in blue) and upregulated in the APAP+UA vs. APAP (in pink). **(C)** Gene Ontology (GO) enrichment analysis of 226 overlapped altered genes showing the 20 regulated terms. **(D)** Kyoto Encyclopedia of Genes and Genomes (KEGG) pathway analysis of 226 overlapped altered genes.

To deliberate the mechanism by which UA protected against ALI, we performed RNA-sequencing analysis to profile the UA-mediated transcriptome profiling in the APAP-induced hepatic injury. There are 2185 genes upregulated and 1882 genes downregulated at least 2-fold change in the APAP challenged mice compared with control (*P*<0.05) (Figure S2A). Kyoto Encyclopedia of Genes and Genomes (KEGG) pathway analysis and Gene Ontology (GO) enrichment analysis of the differentially upregulated genes showed that inflammatory response, apoptosis, and necrosis were included in the top 20 enriched pathways in the APAP mice, confirming the establishment of the APAP-induced ALI model (Figure S2B and C). Furthermore, UA treatment induced 490 genes downregulated, and 290 genes upregulated which were at least 2-fold compared with APAP (*P*<0.05) (Figure 3A). Venn diagram analysis demonstrated that 226 genes were downregulated in APAP *vs* CTL but upregulated in APAP+UA *vs* APAP (Figure 3B). GO and KEGG enrichment analysis of these 226 overlapped differentially expressed genes showed that mitophagy, autophagy, and longevity were enriched in the upregulated signaling pathway in the UA treated mice (Figure 3C and D), consistent with the beneficial role of UA which was previously described 19, 21.

**Figure 4:**
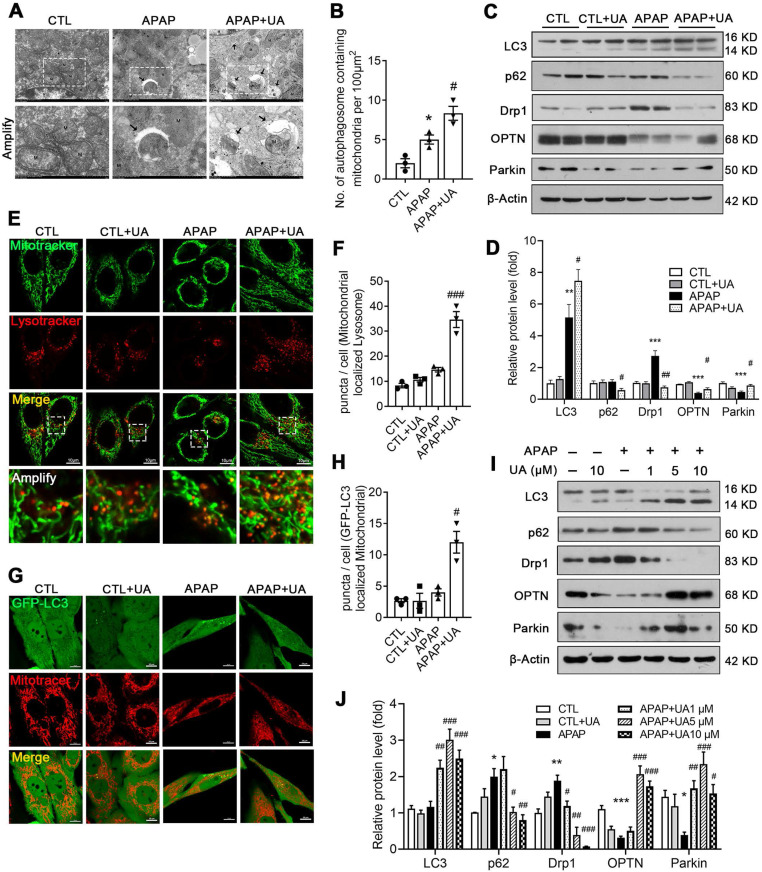
** UA promoted mitophagy after APAP injury *in vivo* and *in vitro*. (A)** Representative images of electron micrographs of hepatic tissue of the mice. Arrows indicate autophagosomes containing mitochondrial fractions. M indicate mitochondria. **(B)** The number of autophagosomes containing mitochondrial fractions per 100 μm^2^ were compared among three groups. **(C)** The protein expression LC3, p62, Drp1, OPTN, and Parkin was detected by immunoblotting, and β-actin was used as the loading control. **(D)** The quantitative densitometric analysis of LC3, p62, Drp1, OPTN, and Parkin proteins expression (*n* = 4).** (E)** L02 cells were treated with or without the treatment of UA (5 μM) in the presence or absence of APAP for 24 h. At the end of experiment, Mitotracker (Green) and Lysotracker (Red) were co-stained. scale bars: 10 μm. **(F)** Quantitative analysis of cells that contained fragmented mitochondria-localized lysosomes from three independent experiments. **(G)** L02 cells were transfected with GFP-LC3 for 36h and then treated with or without the treatment of UA (5 μM) in the presence or absence of APAP for 24 h. At the end of experiment, Mitotracker (Red) was stained and confocal microscopy was used to detect to colocalization of mitochondria and GFP-LC3 puncta, scale bar: 10 μm. **(H)** Quantification of GFP-LC3 puncta that colocalize with Mitotracker per cell from three independent experiments. **(I)** Representative immunoblots and **(J)** analysis of LC3, p62, Drp1, OPTN, and Parkin proteins expression in the L02 cells treated with or without the treatment of UA in the presence or absence of APAP for 24 h. Data were represented as the means ± SEM, three independent experiments for the *in vitro* experiments. ^*^ *P* < 0.05, ^**^ *P* < 0.01, ^***^ *P* < 0.001 *vs.* CTL; ^#^ *P* < 0.05, ^##^ *P* < 0.01, ^###^ *P* < 0.001 *vs.* APAP.

Given that the RNAseq data indicated that UA promotes mitophagy, which is consistent with the previous studies 19, 28, we next investigated whether mitophagy mediated the protective effect of UA against APAP overdose. To this end, electron microscopy assay was performed to observe the mitophagosomes (Figure 4A). Numerous swollen mitochondria with reduced matrix density were observed in the mice with APAP overdose. In contrast, UA treatment significantly increased autophagic vesicles encasing damaged mitochondria, and improved microstructure organization in the APAP-challenged mice (Figure 4A and B). The protein levels of autophagic gene LC3II/LC3I was increased with UA treatment. The autophagic flux marker, P62 (Sequestosome-1) were elevated in the liver of APAP-challenged mice, while significantly attenuated by UA treatment. Drp1, a mitochondrial fission protein, which is closely related to mitochondrial injury 29, was found to be elevated in the APAP-challenged mice. Notably, UA treatment significantly attenuated APAP-induced Drp1 elevation. On the other hand, the mitophagy proteins, Parkin and OPTN were found to decrease after APAP overdose, which was attenuated by UA treatment (Figure 4C and D).

We further examined the role of UA in regulating mitophagy *in vitro*. UA treatment increased both the co-localization of the mitochondrial probe Mitotracker and the lysosomal probe Lysotracker (Figure 4E and F) and the co-localization of GFP-LC3 adenovirus and Mitotracker (Figure 4G and H). In addition, the ratio of LC3II/LC3I and P62 degradation were increased with UA treatment in a dose-dependent manner. Consistent with what we found *in vivo*, APAP-induced Drp1 expression was attenuated by UA treatment. Both Parkin and OPTN were upregulated by UA in a dose-dependent manner (Figure 4I and J). Together, these data indicated that UA activated mitophagy against APAP both *in vivo* and *in vitro*.

### Protection of Urolithin A against AILI is independent of mitophagy

To probe the contribution of mitophagy to UA-mediated protection against APAP, we established hepatocyte-specific Atg5 haplo-insufficient mice, in which mitophagy was impaired 30. We used a cre-loxP-dependent conditional gene targeting approach by crossing Atg5*^flox/flox^* mice with a transgenic mouse expressing hepatic-specific Albumin promoter-driven cre recombinase (Figure S3A). The heterozygous pups with Atg5 haploinsufficiency were selected to investigate the involvement of mitophagy. The control (Alb-cre*^+^*; Atg5*^+/+^*) and Atg5 knockdown (Alb-cre*^+^*; Atg5*^flox/+^*) mice were subjected to APAP challenge, with the vehicle or UA treatment (Figure S3B). There is no detectable difference for both hepatic necrosis area and serum level of ALT or AST in the Atg5 haplo-insufficient mice compared to control mice after the APAP challenge. Unexpectedly, UA treatment still reduced ALT and AST levels in the APAP challenged mice even with hepatic haploinsufficiency of Atg5 (Figure S3C and D). Similarly, the protection of UA against APAP overdose-induced hepatic necrosis was not abated by Atg5 knockdown (Figure S3E). It appeared that although UA activates mitophagy in the murine model of APAP challenge, however, mitophagy did not dominate in mediating the protective action of UA against APAP overdose-induced hepatotoxicity.

### Urolithin A inhibited oxidative stress accumulation and activated Nrf2 pathway

**Figure 5:**
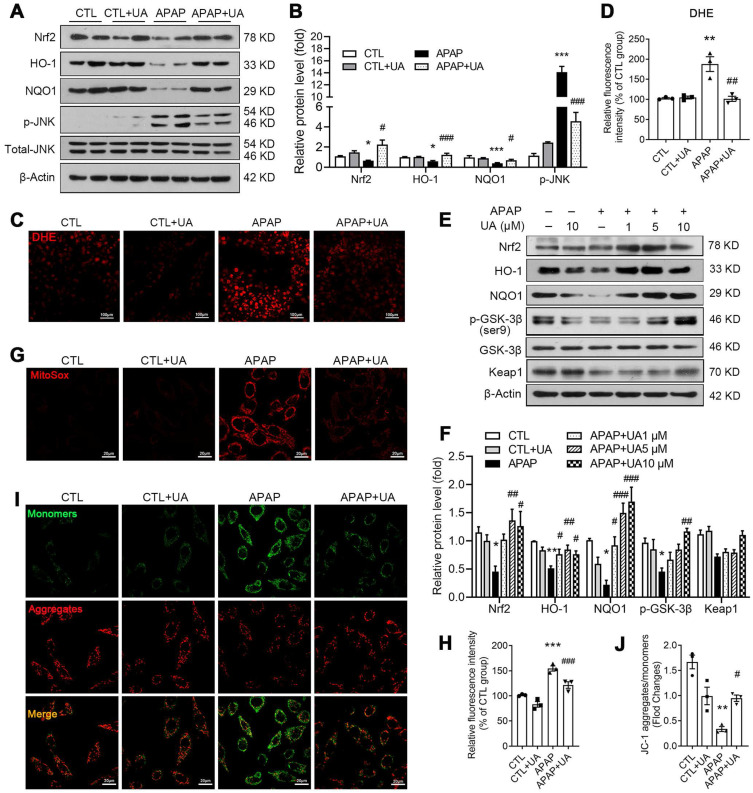
** UA activated Nrf2/ARE signaling and alleviated oxidative stress with the APAP challenge.** L02 cells were treated with or without the treatment of UA in the presence or absence of APAP for 24 h. **(A)** Representative immunoblots and **(B)** analysis of Nrf2, HO-1, NQO1, and p-JNK proteins expression in mice. Data were represented as the means ± SEM, *n* = 6. **(C)** Representative images and **(D)** analysis results of DHE staining in frozen sections of the liver tissue of mice, scale bar: 100 μm. **(E)** Representative immunoblots and analysis **(F)** of Nrf2, HO-1, NQO1, p-GSK-3β, and Keap1 proteins expression in L02 cells. **(G)** Representative images of MitoSox staining in L02 cells, scale bar: 20 μm. **(H)** The quantification of the MitoSox. **(I)** Representative images and analysis **(J)** of JC-1 staining, scale bar: 20 μm. Data were represented as the means ± SEM, the *in vitro* experiment was repeated at least 3 times. ^*^ *P* < 0.05, ^**^ *P* < 0.01, ^***^ *P* < 0.001 *vs.* CTL; ^#^ *P* < 0.05, ^##^ *P* < 0.01, ^###^ *P* < 0.001 *vs.* APAP.

We further analyze the KEGG analysis of RNAseq data and found that Nrf2 signaling was enriched in the UA upregulated genes (Figure 4D). Nrf2, as an important anti-oxidant transcriptional factor 31, was decreased in APAP challenged mice, which was rescued by UA treatment (Figure 5A and B). And its downstream genes including HO-1, NQO1 were significantly increased, confirming that UA treatment activated Nrf2 signaling pathway in the APAP challenged mice. DHE staining showed that the level of oxidative stress in the hepatic tissue section was significantly increased in the APAP-challenged mice, which was significantly attenuated by UA treatment (Figure 5C and D). Consistent with the previous study 32, JNK phosphorylation was increased with APAP challenge, which was also reversed by UA treatment (Figure 5A and B).

Consistent with what we found *in vivo*, UA increased the protein level of Nrf2, HO-1, and NQO1 in a dose-dependent manner (Figure 5E and F) *in vitro*. As shown in Figure 5E and F, UA increased the phosphorylation level of GSK-3β at Ser9 dose-dependently, without affecting Keap1 protein level. We further examined the effect of UA on oxidative stress *in vitro*. MitoSox staining assay demonstrated that mitochondrial superoxide production in L02 cells was increased after APAP challenge, which was attenuated by UA treatment (Figure 5G and H). Urolithin A significantly attenuated the mitochondrial membrane potential loss induced by APAP (Figure 5I and J) by the JC-1 dye test. These results revealed that UA treatment activated Nrf2 signaling pathway and suppressed mitochondrial oxidative stress under APAP challenge.

### Molecular modeling study revealed the binding of UA with Nrf2

**Figure 6:**
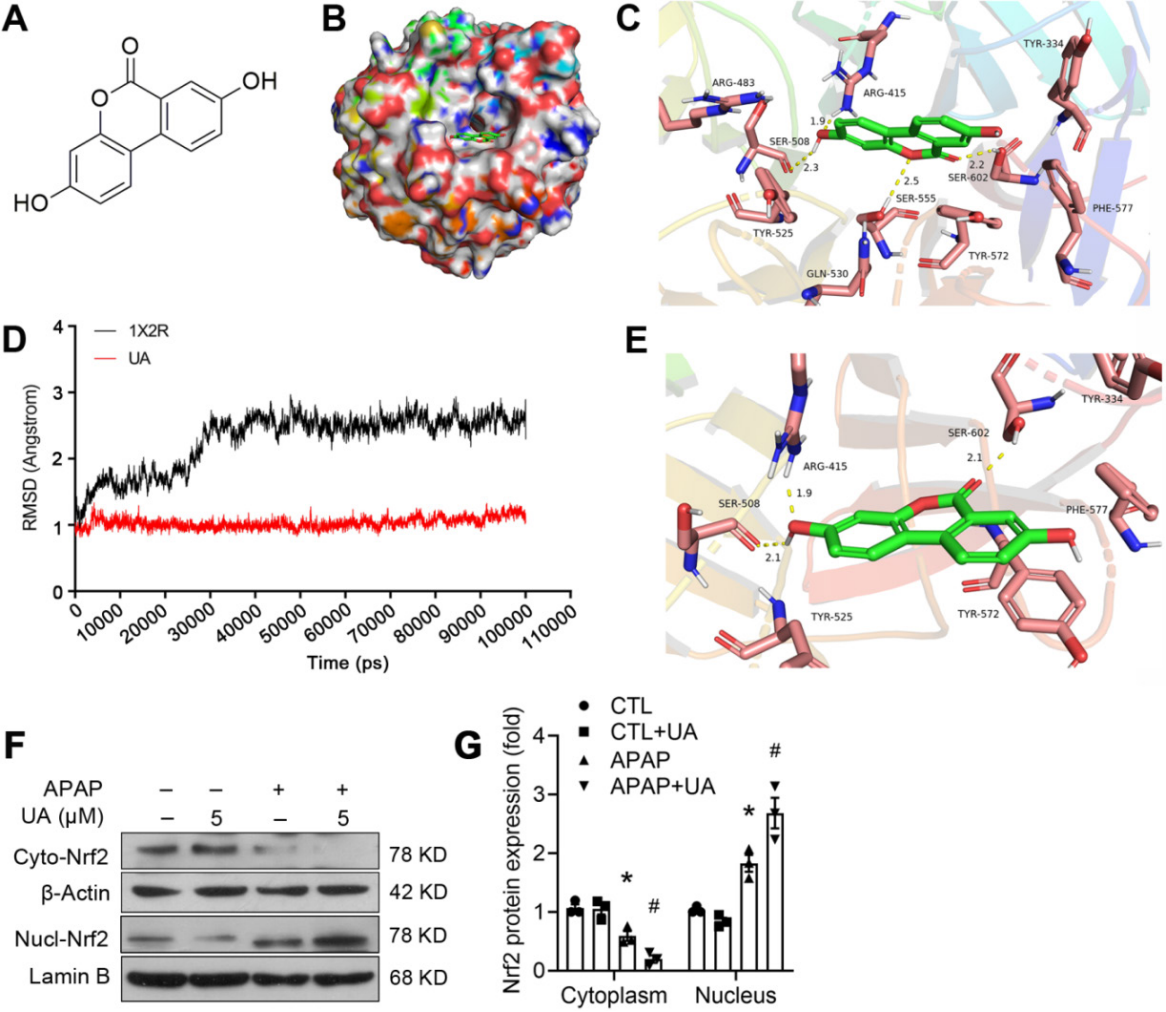
**A molecular modeling study revealed the binding of UA with Nrf2. (A)** The structure of UA. **(B)** Surface representation of the crystal structure of Keap1 (PDB ID: 1X2R) in complex with UA (green). **(C)** The binding mode of Keap1 with UA. H-bond (yellow): Arg 415 (1.9 Å), Ser 508 (2.3 Å), Ser 555 (2.5 Å), Ser 602 (2.2 Å). **(D)** RMSD values investigation through MD simulations. **(E)** The last frame was extracted as a representative. **(F)** Representative immunoblots and analysis **(G)** of cyto- and nuclear-Nrf2. Data were represented as the means ± SEM, the *in vitro* experiment was repeated at least 3 times. ^*^ *P* < 0.05, *vs.* CTL; ^#^ *P* < 0.05 *vs.* APAP.

To clarify whether there is a direct binding of UA to the Nrf2, we further performed molecular docking using Discovery Studio 3.1 software (Fig. 6A-C). The binding mode revealed that UA binds the pocket of Nrf-2/Keap1 complex (PDB ID:1X2R) by 4 H-bond bindings: Arg 415 (1.9 Å), Ser 508 (2.3 Å), Ser 555 (2.5 Å), Ser 602 (2.2 Å). Moreover, based on the complex model attained from molecular docking, a 100 ns MD simulation was conducted to investigate the dynamic traits of Nrf2/UA complex. The time evolution of weighted RMSDs for backbone atoms of the Nrf2 protein and UA from their original positions (t=0) was obtained to calculate the structural stability of the complex in MD simulation. As shown in Fig. 6D-6E, RMSD values of the protein backbone ranged from 0.76Å to 1.53 Å during the process of MD simulations. The stable RMSD values for UA and the protein's heavy atoms confirmed that the system is well-equilibrated. A well-defined substrate pocket was demonstrated by the analyses of H-bond and hydrophobic interaction. As shown in Fig. 6E, there are 3 conserved H-bonds between UA and Nrf2 in the complex (Ser 508, Arg 415, Ser 602), which is consistent with the docking results. Furthermore, UA significantly decreased the cytoplasmic expression but increased the nuclear accumulation of Nrf2 (Figure 6F and G), implying that the extensive polar interactions exist between UA and the Nrf-2/Keap1 residues, which may in turn trigger Nrf2 nuclear translocation and leads to the activation of Nrf-2 target genes.

### Nrf2 Knockdown blunted the protective effect of UA in APAP-induced liver injury

**Figure 7:**
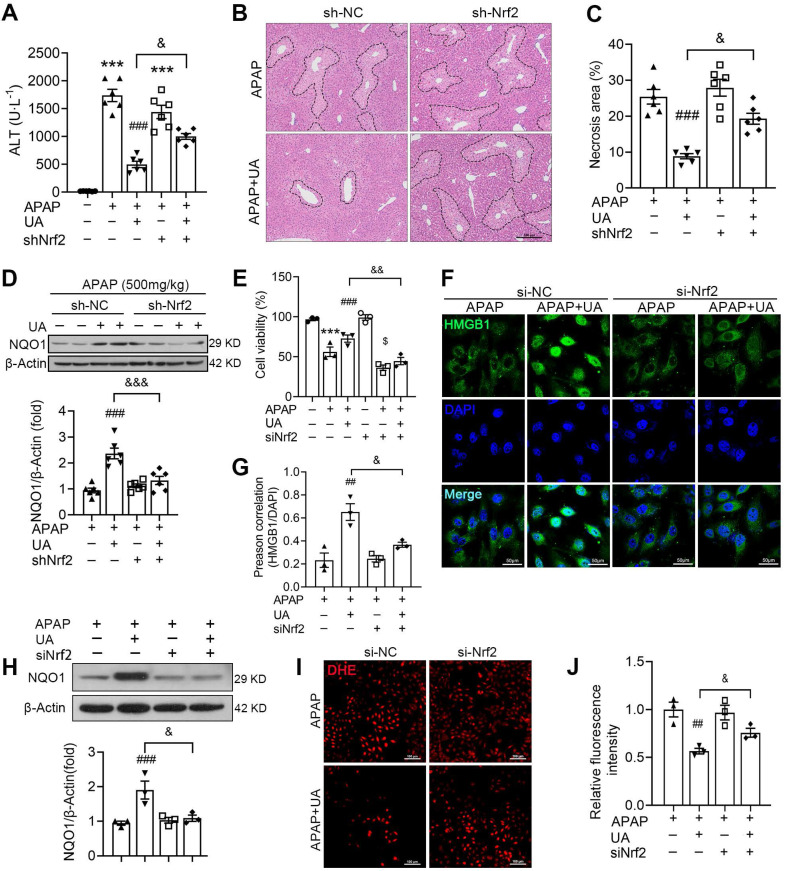
** UA alleviates APAP-induced oxidative stress through Nrf2 signaling pathway.** The mice were pretreated with AAV8-sh-Nrf2 (5 × 10^10^ vg/mL) or Scramble shRNA via tail vein injection. One week after AAV infection, the mice were challenged with APAP injection with or without UA treatment (50 mg/kg). The liver tissue and serum were harvested at 12 h after APAP challenge. *n* = 6 mice per group. **(A)** Serum ALT levels from different groups. **(B)** Representative images of hematoxylin and eosin-stained liver sections, scale bar: 100 μm. **(C)** The quantification of necrosis area of the liver tissue. **(D)** Representative immunoblots and analysis of NQO1 expression in mice were treated with or without Nrf2 shRNA. **(E)** The L02 cells were transfected with the siNrf2 RNA or Scramble siRNA for 48 h and then treated with or without UA (5 μM) in the presence or absence of APAP (10 mM) for 24 h. Cell viability was determined by CCK8 assay. **(F)** HMGB1 immunofluorescence of L02 cells. scale bar: 50 μm. **(G)** Quantification of colocalization of HMGB1 and nuclei. **(H)** Representative immunoblots and analysis of NQO1 expression in L02 cells. **(I)** Representative images of DHE staining of L02 cells, scale bar, 100 μm. **(J)** The quantification of the DHE positive intensity. Data are expressed as mean ± SEM. The *in vitro* experiment was repeated at least 3 times. ^***^ *P* < 0.001 *vs.* CTL; ^##^ *P* < 0.01, ^###^ *P* < 0.001 *vs.* APAP; ^&^* P* < 0.05, ^&&&^* P* < 0.001 *vs.* siNC-APAP + UA or sh-NC-APAP + UA.

Nrf2/ARE system in the protective effect of UA against acetaminophen hepatotoxicity, we silenced Nrf2 by tail vein injection of an AAV8-sh-Nrf2. The expression of Nrf2 in mice was measured by western blot to verify Nrf2 knockdown successfully (Figure S4A). As shown in Figure 7A, the therapeutic effect of UA on acetaminophen hepatotoxicity was mitigated by Nrf2 knockdown, as revealed by serum ALT level. Moreover, histological assessment demonstrated that the hepatic necrosis area in the APAP challenged mice with UA treatment was increased in the Nrf2 knockdown mice compared with that with sh-NC control (Figure 7B and C). The protein level of NQO1 was significantly inhibited with sh-Nrf2 even with UA treatment (Figure 7D).

In addition, we examined whether Nrf2 knockdown was involved in the protective effect of UA *in vitro*. Western blot assay showed that Nrf2 was successfully knocked down by Nrf2 siRNA (Figure S4B). The cell viability assays showed that the protective effect of UA against acetaminophen hepatoxicity was blunted with siNrf2 (Figure 7E). Furthermore, siNrf2 abolished UA-mediated HMGB1 nuclearization with acetaminophen challenge (Figure 7F and G). Of note, siNrf2 significantly inhibited UA-induced activation of its downstream target, NQO1 (Figure 7H). And UA-mediated ROS suppression was also mitigated by siNrf2 (Figure 7 I and J).

Collectively, these data from *in vivo* and *in vitro* experiments illustrated that Nrf2/ARE signaling activation participates in the protective effect of UA against acetaminophen hepatotoxicity.

### Urolithin A exerts better therapeutic Effects on AILI than NAC

**Figure 8:**
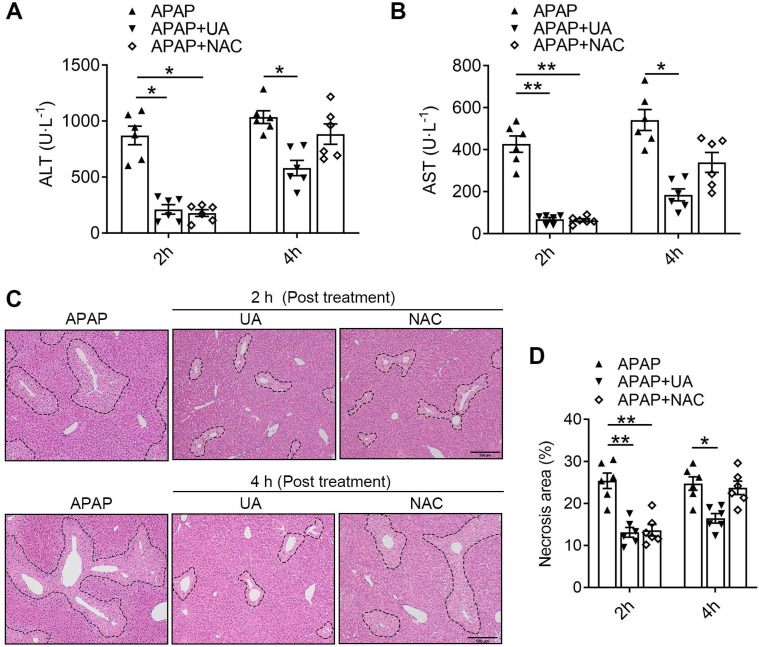
** UA has an extended therapeutic window for APAP-induced liver injury compared with NAC.** The mice received UA (50 mg/kg, i.p.) or NAC (300 mg/kg, i.p.) at 2 h or 4 h after acetaminophen overdose. Serum and liver tissues were collected 12h after APAP injection **(A)** Serum ALT levels. **(B)** Serum AST levels. **(C)** Representative images of H&E-stained liver sections at 100 × magnification, scale bar: 100 μm. **(D)** Quantification of necrosis area of the liver tissue. Data are presented as mean ± SEM for n = 6 mice per group. ^*^ *P* < 0.05 *vs.* APAP; ^**^ *P* < 0.01 *vs.* APAP.

NAC, as the only therapeutic drug for acetaminophen overdose in clinic, was limited due to the finite time window 8, 9. To explore the potential translational significance, we compared the therapeutic effects of UA against APAP toxicity with that of NAC. We found that 2 h post-treatment of both UA and NAC significantly lowered ALT and AST levels in the acetaminophen challenged mice (Figure 8A and B). However, the 4 h post-treatment with NAC lost its protective effect, as revealed by serum ALT and AST levels. In contrast, UA is still protective in post-treatment at 4 h after APAP administration. Furthermore, UA at a dosage of 50 mg/kg led to a much higher decrease of serum ALT and AST in contrast to NAC at a dosage of 300 mg/kg. Moreover, the histological assessment confirmed that NAC is only effective within 2 h post-treatment after APAP challenge, however, UA is still effective until 4 h post-treatment (Figure 8C and D). These data suggest that UA is superior on AILI with better potency and therapeutic time window compared to NAC.

## Discussion

APAP overdose-induced hepatotoxicity is the most common trigger of acute liver failure worldwide including the United States. Unfortunately, the therapeutical options for this fatal disease are rather limited. Here, we unveiled that a gut microbiota metabolite of ellagitannins, UA is potentially protective against APAP-induced hepatoxicity both *in vivo* and *in vitro* through activating Nrf2/ARE signaling pathway, which alleviates oxidative stress and mitochondrial dysfunction. Notably, UA is superior to the only standard antidote drug NAC in terms of dosage and therapeutic time windows.

UA was identified as a potential mitophagy inducer and was found protective in multiple aging-related conditions 19-21. Besides the effect on mitophagy inducer, UA was identified as a direct radical scavenger, which could contribute to its protective properties 33. We previously demonstrated that urolithin B, another metabolite of ETs, could reduce ischemia/reperfusion-induced oxidative stress in an Nrf2‐dependent manner 25. Here we firstly demonstrated that UA treatment protect against APAP overdose-induced liver injury, as evidenced by decreased serum ALT and AST, and hepatic necrosis. Although there is no direct evidence of UA protecting the liver, a couple of investigations suggest that UA plays an essential role in regulating metabolism, insulin resistance, and obesity, which could help to improve liver hemostasis 34-36. For example, UA was found to significantly improve insulin resistance, reduce hepatic triglycerides accumulation, and alleviate hepatic steatosis in high fat die-fed mice 36. As such, our data provides the first direct evidence of a hepatic protective effect of Urolithins in APAP-overdose challenge. Further studies are warranted to extend our concept to other types of hepatic disease models and the potential clinical applications of Urolithins.

Mitophagy refers to the autophagic removal of damaged mitochondria via a selective autophagy process. It has been reported that mitophagy is activated by acetaminophen overdose and acts as an adaptive mechanism by removing acetaminophen-protein adducts and damaged mitochondria 37. Mitophagy deficiency via PINK1/Parkin double deletion further elevated serums of ALT and mortality after APAP challenge 38, further suggesting mitophagy plays a critical role via dissolving damaged mitochondria. Given that UA is a mitophagy inducer, we hypothesize that UA may exert a hepatic protective effect via mitophagy activation. Consistent with the previous studies 19, UA stimulates mitophagy both *in vivo* and *in vitro*, evidenced by the increased number of mitophagosomes and colocalization of GFP-LC3 and Mitotracker. Knockdown of Atg5 interfered autophagosome formation and mitophagy, inhibiting subsequent mitochondrial removal by mitophagy 39, 40. Unexpectedly, in the hepatocyte-specific Atg5 knockdown mice, the hepatoprotective effect of UA still occurs, precluding the role of mitophagy activation in the effect of UA against APAP overdose-induced hepatotoxicity. We cannot exclude other additional compensatory mechanisms of the UA-mediated protective effect in wild type and Atg5 hepatic deficient animals after APAP overdose challenge.

Previous studies demonstrated that urolithins, as a class of antioxidant polyphenols, protected against oxidative stress in multiple organs such as heart, liver, and kidney 15, 41. Along this line, our results showed that UA activated the Nrf2/ARE singling pathway and alleviated oxidative stress and mitochondrial dysfunction. Nrf2, as a transcriptional factor, regulates a large board of genes through the antioxidant response element (ARE) binding sites on the promoter regions 42. In line with our observations, activating Nrf2/ARE signaling pathway ameliorates liver injury caused by a high dose of APAP 43, 44. And antioxidants targeting Nrf2 signaling pathway have been shown to ameliorate AILI 44, 45. Consistent with our findings, Nrf2 was identified as a negative downstream target of p-JNK, which provides hepatic defense against APAP toxicity via ARE-driven gene expression and anti-oxidant response 44.

Although Keap1, a negative physiological inhibitor of Nrf2, is widely believed to be essential in regulating Nrf2 protein stability through the cullin3 ubiquitination pathway^46^-^48^, we did not detect the inhibition of UA on Keap1. We speculated that the direct binding of UA with Nrf-2/Keap1 residues Arg415, Ser508 and Ser602, which in turn trigger Nrf2 nuclear translocation, subsequently leading to activation of Nrf-2 target genes (HO-1, NQO1). In our study, although UA did not alter the expression level of Keap1 protein, we cannot exclude the possibilities that UA modify the interaction between Nrf2 and Keap1 by entering the the pocket of Nrf2-Keap1 complex, without changing the Keap1 protein level. Additionally, the regulation of Nrf2 through release from the Keap1-Nrf2 complex is usually is rapid and transient 49, which cannot elucidate the sustained activation of Nrf2 and its downstream target genes for 12 h after APAP challenge. A couple of Keap1-independent Nrf2 degradation mechanisms including GSK-3β-TrCP pathway have been described 46, 50. In our study, UA treatment induced a significant increase of inactive *p*-GSK-3β (S9) in the APAP-challenged hepatocytes. It was demonstrated that *p*-GSK-3β (S9) releases Nrf2 from β-TrCP-mediated degradation complex 51. Whether UA is a GSK-3β modulator itself or there is additional molecular regulation, requires further investigation.

NAC, as the only standard antidote for acetaminophen toxicity approved by FDA, is effective at the early stage, however, rather limited to the therapeutic window. And NAC treatment has to be assigned by the serum APAP level. Actually, the APAP is undetectable in more than 50% of patients who present at the hospital with APAP hepatotoxicity, which usually exceeds the rescue time window, and even dismisses the possibility of NAC treatment 52. As such, medications with long time-frame would be greatly favorable. In mice, hepatic damage usually initiates at 3 h and peaks around 6 h post-APAP 53. UA is effective as long as 4 h post-APAP, longer than NAC, indicating UA hold promising potential for late-presenting patients in this setting. Furthermore, A recent randomized, double-blind, placebo-controlled clinical study demonstrated that UA daily supplementation in healthy elderly for 4-week is favorably safe, bioavailable, and displayed improved mitochondrial function and cellular health 21, which enhanced the translational significance of UA in humans 21, 54. Moreover, we found that UA triggered a sustained activation of Nrf2, which was even obvious at 12 h post-APAP-challenge, a time point when the oxidative stress had already developed 55, 56. Although the precise mechanisms by which UA displays a superior therapeutic time window remains elusive, we observed a better therapeutic property of UA against APAP overdose-induced hepatotoxicity in contrast to NAC, as evidenced by a lower therapeutic dosage and longer time window.

In summary, our result demonstrated that UA, a natural occurring metabolite of ETs with proven biosafety 21, 54, alleviated acetaminophen-induced hepatotoxicity via sustained activation of Nrf2/ARE signaling, which could suppress acetaminophen overdose-induced oxidative stress, thereby protecting against AILI. Given the remarkable hepatoprotective effect of UA, our data provide constructive insights into potential therapeutic strategies to treat APAP overdose.

## Supplementary Material

Supplementary figures.

## Figures and Tables

**Scheme 1:**
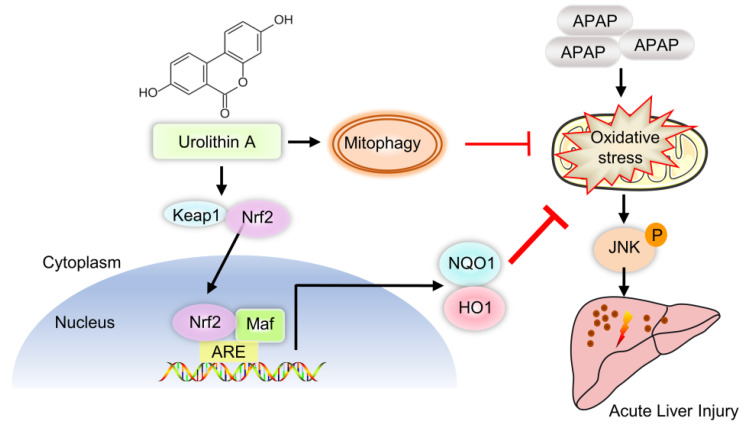
** Schematic diagram of Nrf2/ARE signaling and mitophagy mediated by UA treatment against ALI.** UA, a metabolite of ellagitannin natural products in the gastrointestinal flora, alleviated acetaminophen-induced oxidative stress and hepatic necrosis via activating Nrf2/ARE signaling pathway rather than mitophagy activation. Mechanistically, UA interacted with Nrf-2/Keap1 complex, which in turn trigger Nrf2 nuclear translocation, subsequently leading to activation of Nrf-2 target genes (HO-1, NQO1), followed by suppression of oxidative stress and JNK phosphyralation, thereby protecting against AILI.
