# Supplementary material for: Urolithin A protects against acetaminophen-induced liver injury in mice via sustained activation of Nrf2
Source: Int J Biol Sci. 2022 Feb 28;18(5):2146–62. doi: 10.7150/ijbs.69116 (PMC8935220; doi:10.7150/ijbs.69116)
Supplement: Supplementary file 1 — Supplementary figures. [file ijbsv18p2146s1.pdf]

Supplementary Figure legends

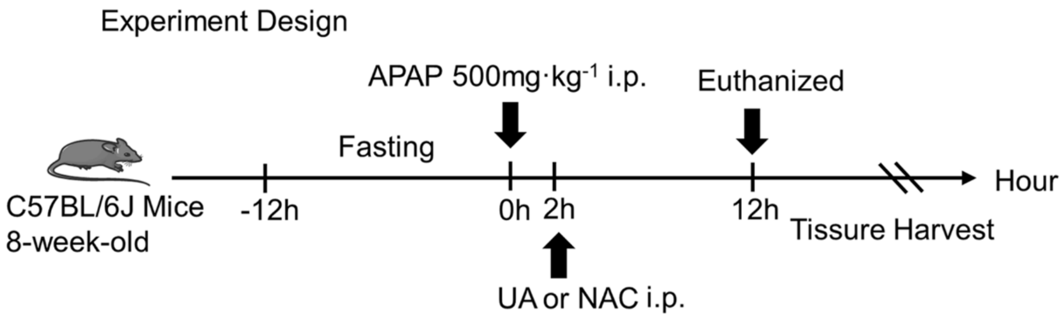

Figure S1. Experimental design to examine the effects of UA in APAP-induced liver injury. UA (50, 100, 150 mg/kg) or NAC (300 mg/kg) was administered intraperitoneally to mice after APAP (500 mg/kg) injection.

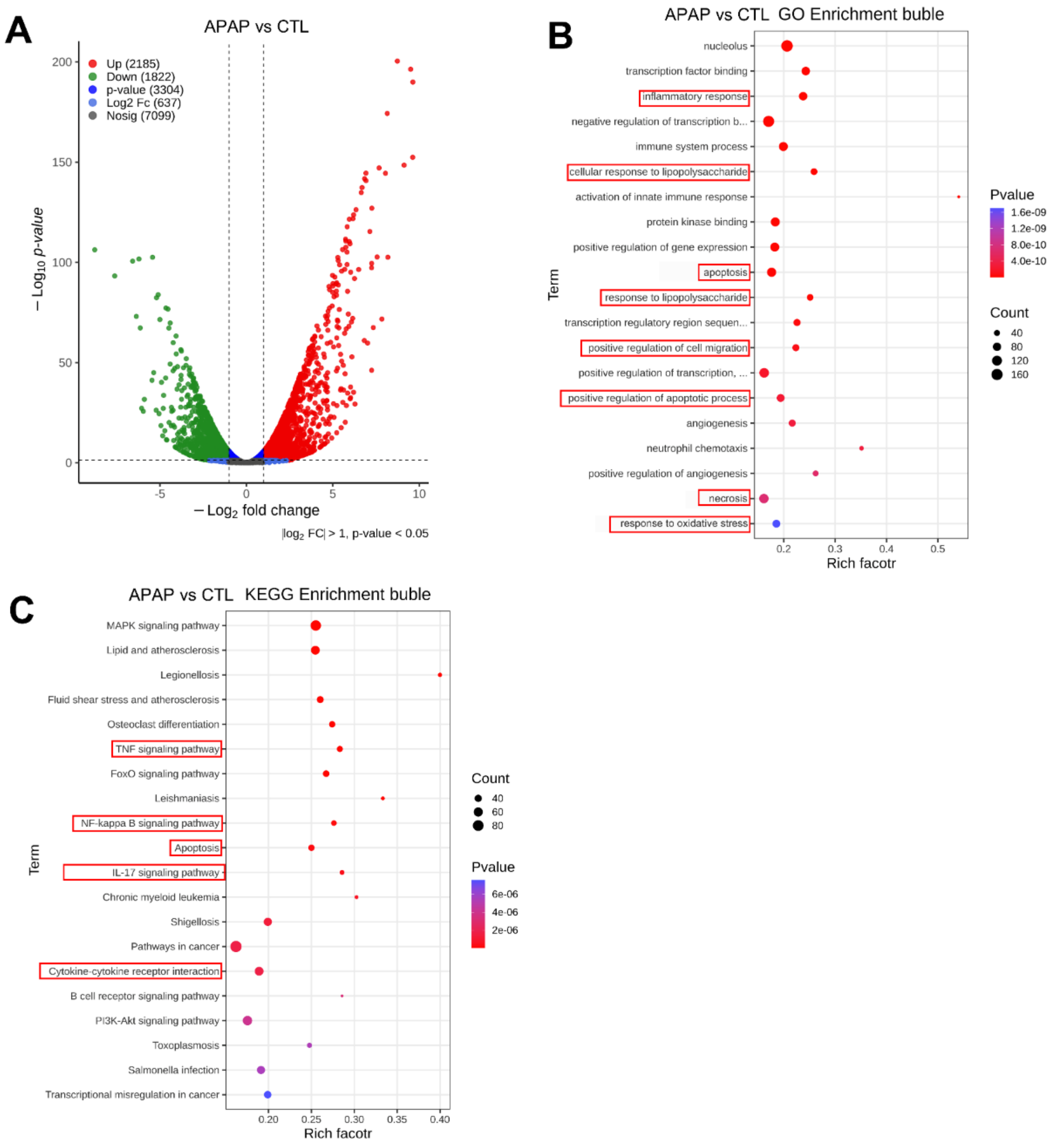

Figure S2. RNA-Seq identifies transcriptome that are dysregulated in the APAP-challenged liver tissue. (A) Volcano plot shows the differentially expressed genes in the liver after APAP challenge. Genes of downregulation (left) and upregulation (right) in APAP versus CTL were plotted in green and red dot, respectively. **(C)** Gene Ontology (GO) enrichment analysis of the upregulated genes showing the top 20 regulated terms in the APAP liver versus CTL. **(D)** Kyoto Encyclopedia of Genes and Genomes (KEGG) pathway analysis of the upregulated genes.

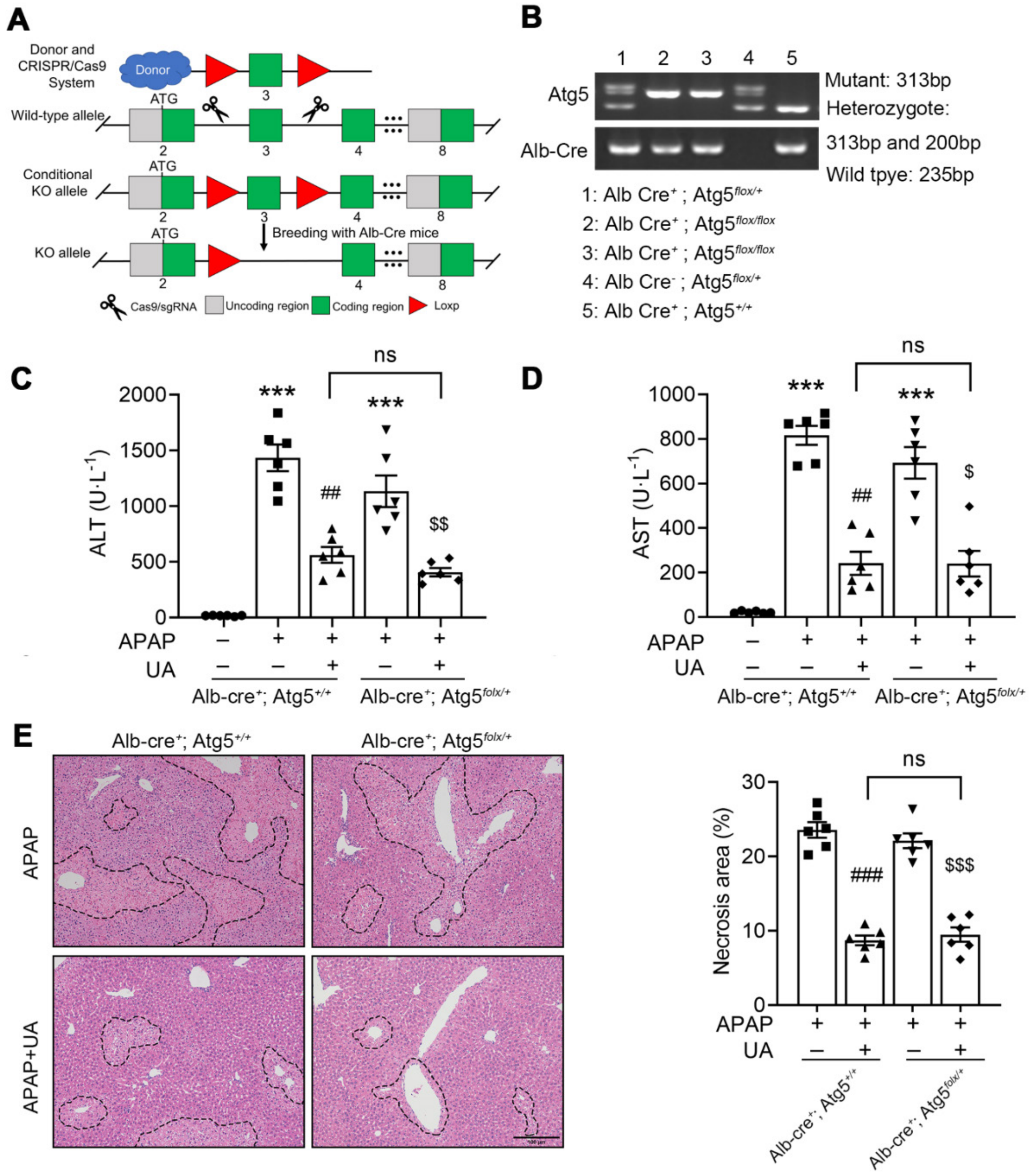

Figure S3. UA protected against APAP-induced liver injury is independent of mitophagy. The wild-type and Alb-Cre$^+$; Atg5 $^{flox/+}$, mice were administered with APAP (500 mg/kg, i.p.) or saline for 12 h with or without UA (50 mg/kg) treatment (*n* = 6). **(A)** Schematic strategies for the generation of Atg5 haploinsufficient mice using CRISPR/Cas9 technology. **(B)** The genotype of each mouse was confirmed with PCR. **(C)** Serum ALT and **(D)** AST levels in mice. **(E)** Representative image and analysis of histological sections stained with H&E of the liver, scale bar: 100 μm. $^{***}P$<0.001 *vs.* Alb-Cre$^+$; Atg5 $^{+/+}$ without APAP; $^{\#\#}P$<0.01 *vs.* Alb-Cre$^+$; Atg5 $^{+/+}$mice without UA

treatment; $^{\$}P< 0.05$, $^{\$\$}P< 0.01$, $^{\$\$\$}P< 0.001$ *vs.* Alb-Cre^{+}; Atg5 $^{flox/+}$mice without UA treatment, ns: no significance *vs.* Alb-Cre^{+}; Atg5 $^{+/+}$ with UA treatment, $n = 6$.

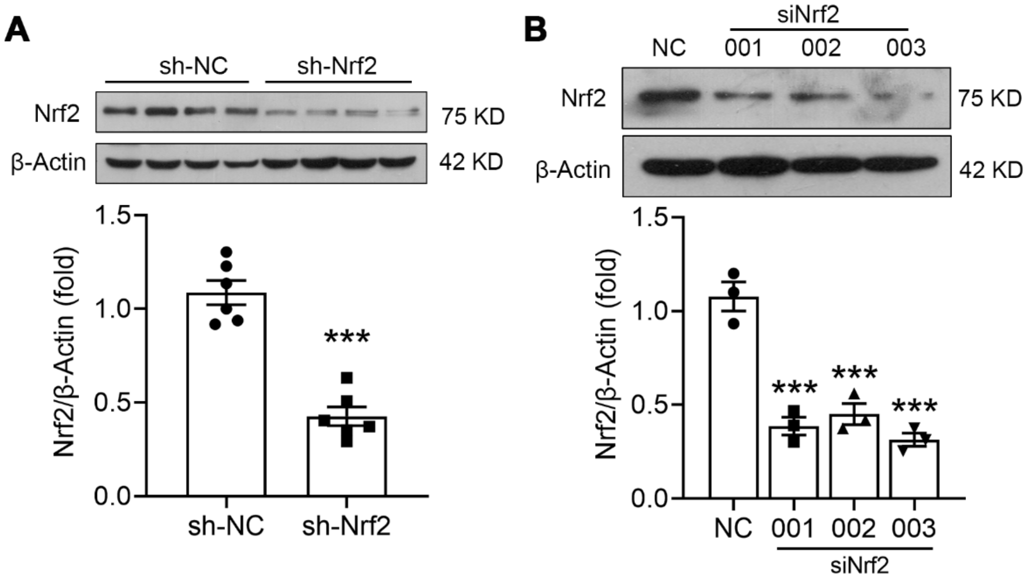

Figure S4. Verification of Nrf2 knockdown *in vivo* and *in vitro*. (A) Immunoblots of Nrf2 protein expression in the liver tissue of the mice with sh-Nrf2 or sh-NC. **(B)** Immunoblots of Nrf2 protein expression in L02 cells with si-Nrf2 or si-NC. *** P < 0.001 *vs*. NC, n = 3.
